# Tree Regeneration After Unprecedented Forest Disturbances in Central Europe Is Robust but Maladapted to Future Climate Change

**DOI:** 10.1111/gcb.70734

**Published:** 2026-02-06

**Authors:** Mária Potterf, Christian Schattenberg, Kirsten Krüger, Kilian Hochholzer, Werner Rammer, Marc Grünig, Kristin H. Braziunas, Christina Dollinger, Aikio Erhardt, Jean‐Claude Gégout, Lisa Geres, Sina Greiner, Tomáš Hlásny, Anne Huber, Jonas Kerber, Judit Lecina‐Diaz, Lisa Mandl, Roman Modlinger, Johannes S. Mohr, Jörg Müller, Miguel Muñoz Mazón, Paulina E. Pinto, Tobias Richter, Sebastian Seibold, Cornelius Senf, Josep M. Serra‐Diaz, Ana Stritih, Dominik Thom, Alba Viana‐Soto, Jiayun Zou, Rupert Seidl

**Affiliations:** ^1^ Faculty of Forestry and Wood Sciences Czech University of Life Sciences Prague Czech Republic; ^2^ Ecosystem Dynamics and Forest Management Group, School of Life Sciences Technical University of Munich Freising Germany; ^3^ Department of Epidemiology and Public Health Swiss Tropical and Public Health Institute (Swiss TPH) Allschwil Switzerland; ^4^ University of Basel Basel Switzerland; ^5^ School of Environmental and Forest Sciences University of Washington Seattle Washington USA; ^6^ Université de Lorraine, AgroParisTech, INRAE, SILVA Nancy France; ^7^ Berchtesgaden National Park Berchtesgaden Germany; ^8^ Earth Observation for Ecosystem Management, School of Life Sciences Technical University of Munich Freising Germany; ^9^ Bavarian Forest National Park Grafenau Germany; ^10^ Department of Animal Ecology and Tropical Biology, Biocenter, Field Station Fabrikschleichach University of Würzburg Rauhenebrach Germany; ^11^ Harvard Forest Harvard University Petersham Massachusetts USA; ^12^ Forest Zoology TUD Dresden University of Technology Dresden Germany; ^13^ Botanical Institute of Barcelona (CSIC‐CMCNB) Barcelona Spain; ^14^ Faculty of Environment and Natural Resources University of Freiburg Freiburg Germany; ^15^ Institute of Silviculture and Forest Protection TUD Dresden University of Technology Dresden Germany

**Keywords:** climate suitability, ecosystem reorganization, forest resilience, hotter drought, maladaptation risk, post‐disturbance recovery

## Abstract

Central Europe has been a hotspot of forest disturbance during 2018–2020, with large pulses of tree mortality from drought and bark beetles. Post‐disturbance recovery is crucial for forest resilience and the continued provision of ecosystem services. We surveyed 849 plots in disturbance hotspots across 10 Central European countries to assess the state of early (3–5 years) post‐disturbance tree regeneration. Our specific objectives were to quantify post‐disturbance tree recovery, identify key drivers, and assess future trajectories using model‐based analyses. We found robust tree recovery throughout Central Europe, with median stem densities of 4750 *n* ha^−1^. Only 7% of plots had no regeneration. Regeneration density increased with precipitation, particularly at warm sites, and decreased with disturbance severity and size. The most frequently regenerating tree species was *Picea abies* (present on 48% of plots), a species that is poorly adapted to future heat and drought. Overall, we found that 75% of the currently established trees are projected to be outside of their climatic niche by the end of the century under moderate climate change (RCP4.5). We conclude that while Central European forests recover well from recent disturbances, they lack sufficient post‐disturbance reorganization to enable sufficient adaptation to future climate.

## Graphical abstract

Central European forests became hotspots of tree mortality from drought and bark beetles between 2018 and 2020, raising concerns about forest recovery. Based on field surveys from 849 disturbed sites in ten European countries, we found that forests are regenerating quickly, with new trees present on most sites within a few years, especially under warm and wet conditions. However, many newly established trees—such as Norway spruce—are poorly suited to future heat and drought conditions. While Central European forests recover well, today's recovery may not ensure long‐term adaptation to future climate.

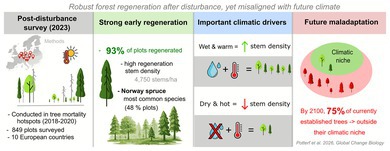

## 1 Introduction

Forest disturbances are increasing worldwide, with their frequency and severity projected to rise further under ongoing climate change (Patacca et al. 2022). This trend raises concerns about forest resilience—the ability of a forest to return to a state that is structurally and functionally equivalent to its pre‐disturbance state following disturbance (Holling 1973). Changing climate and disturbance regimes may push ecosystems beyond their resilience threshold, resulting in lasting changes to forest structure and composition, or even leading to regeneration failure and conversion to non‐forest states (Lenton et al. 2023; Munson et al. 2018). The reorganization window immediately following a disturbance is a particularly crucial phase in the context of resilience (Seidl et al. 2024; Seidl and Turner 2022). This phase of early tree establishment strongly influences forest development for decades and is the period during which ecosystems are particularly sensitive to diverging post‐disturbance trajectories, for example, from positive‐feedback switches (Wilson and Agnew 1992).

Forest recovery after disturbance is a key component of resilience. Central European forests have historically been considered highly resilient systems (Leuschner and Ellenberg 2017), and this notion is supported by evidence from field surveys (Cerioni et al. 2024; Seidl et al. 2024) and remote sensing assessments (Mandl et al. 2024; Senf and Seidl 2022). However, the recent wave of drought‐ and heat‐driven disturbances has been unprecedented in scale and intensity, raising concerns about the success of post‐disturbance recovery under the newly emerging climate and disturbance regimes (Orrock et al. 2023). Specifically, the 2018–2020 disturbance pulse in Europe was the largest in the past at least 170 years, with disturbance rates, sizes and aggregation levels considerably exceeding previously observed maxima (Senf et al. 2018). Hotter drought caused direct tree mortality (Schuldt et al. 2020) and triggered a widespread bark beetle outbreak that was unprecedented in extent and severity for the region (Hlásny et al. 2021; Potterf et al. 2025). Consequently, the fate of disturbed areas has been discussed intensively in forestry and society, and many countries issued policies to aid post‐disturbance recovery. Yet, a systematic assessment of the post‐disturbance state of forests and their reorganization trajectories remains missing to date.

Conditions for post‐disturbance tree regeneration may become increasingly challenging, particularly in regions experiencing prolonged droughts or on exceedingly large disturbance patches. These conditions could severely hamper tree establishment and survival (Serra‐Diaz et al. 2016), raising concerns about regeneration failure and long‐term forest loss (Rammer et al. 2021). Post‐disturbance regeneration success is strongly shaped by environmental conditions and seed availability. Warmer and sufficiently moist conditions generally enhance seed germination, early root growth, and seedling survival, whereas low temperatures and/or water limitation reduce establishment probabilities and increase seedling mortality (Fisichelli et al. 2014; Hansen et al. 2018). In addition, tree regeneration following large disturbance patches is often dispersal‐limited because seed rain declines rapidly with distance from the edge of the undisturbed forest, and low seed input strongly constrains stem densities in the years after disturbance (Orrock et al. 2023). In this context, recent studies have distinguished two distinct patterns of post‐disturbance tree regeneration in Central Europe: advanced and delayed regeneration (Hansen et al. 2018; Petrovska et al. 2023; Seidl et al. 2024; Szwagrzyk et al. 2018). Sites with ‘advanced’ regeneration are characterized by a high density of saplings already early after disturbance (Seidl et al. 2024), which established before drought and/or bark beetles killed the trees in the overstory. Advanced regeneration can considerably accelerate post‐disturbance recovery (Franklin et al. 2007; Szwagrzyk et al. 2018). However, it often consists of the same tree species that dominated the stand prior to disturbance (Kramer et al. 2014), potentially leading to a lock‐in of the tree species composition (Johnstone et al. 2010) and forests that are maladapted to the emerging climate conditions (e.g., heat‐ and drought‐sensitive *Picea abies* , which was often dominant in the overstory prior to the recent disturbance pulse in Central Europe, but may no longer be climatically suitable throughout the 21st century at many sites). In contrast, areas with ‘delayed’ regeneration have no tree establishment for several years following disturbance (Hansen et al. 2018). While regeneration windows can extend over many years in forest ecosystems (Donato et al. 2016; Serra‐Diaz et al. 2018), delays in tree establishment can indicate the presence of limiting factors that could eventually lead to regeneration failure. Currently, the prevalence of advanced and delayed regeneration in Central Europe remains largely unknown. However, a better quantitative understanding of post‐disturbance reorganization is of high relevance for forest policy and management, as the current regeneration determines the future structure and composition of forest ecosystems, and hence also their capacity to provide ecosystem services to society (Lecina‐Diaz et al. 2024).

Here, we (i) analyzed patterns of early recovery (i.e., 3–5 years after disturbance) of temperate forests in Central Europe following the largest pulse of disturbance in recent history (2018–2020 [Hermann et al. 2023]), (ii) quantified the drivers of early post‐disturbance recovery and (iii) assessed potential near‐term (next 30 years) forest development trajectories and long‐term (end of the century) climate suitability of the emerging forests. In line with previous assessments of post‐disturbance forest regeneration based on remote sensing (Senf and Seidl 2022) and field data (Cerioni et al. 2024), we hypothesized that Central European forests are highly resilient and recover swiftly also from the recent wave of disturbances (H1). We further expected that warmer and wetter climatic conditions and sites located closer to the edge of a disturbance patch (and hence near a potential seed source) will have higher stem densities in the early stages of recovery (H2). We additionally contrasted plots with delayed versus advanced regeneration to better understand the prevalence of different post‐disturbance reorganization patterns. Specifically, we hypothesized that advanced regeneration (defined as sites with > 1000 stems ha^−1^ that are > 2 m in height and hence beyond the influence of browsing) dominates post‐disturbance recovery in Central Europe (Petrovska et al. 2023; Seidl et al. 2024; Szwagrzyk et al. 2018). Alternatively, recent extreme drought years may have had a strong negative effect on tree establishment and early growth (Thom et al. 2023), resulting in a substantial area experiencing delayed regeneration (here defined as no trees in the regeneration layer 3 to 5 years after disturbance). Lastly, we expected that a considerable share of the currently established new cohort of trees is poorly adapted to climate change and will be outside of their climatic niche under the conditions expected for the end of the century (Wessely et al. 2024) (H3).

## 2 Materials and Methods

### 2.1 Study Plots

**FIGURE 1:**
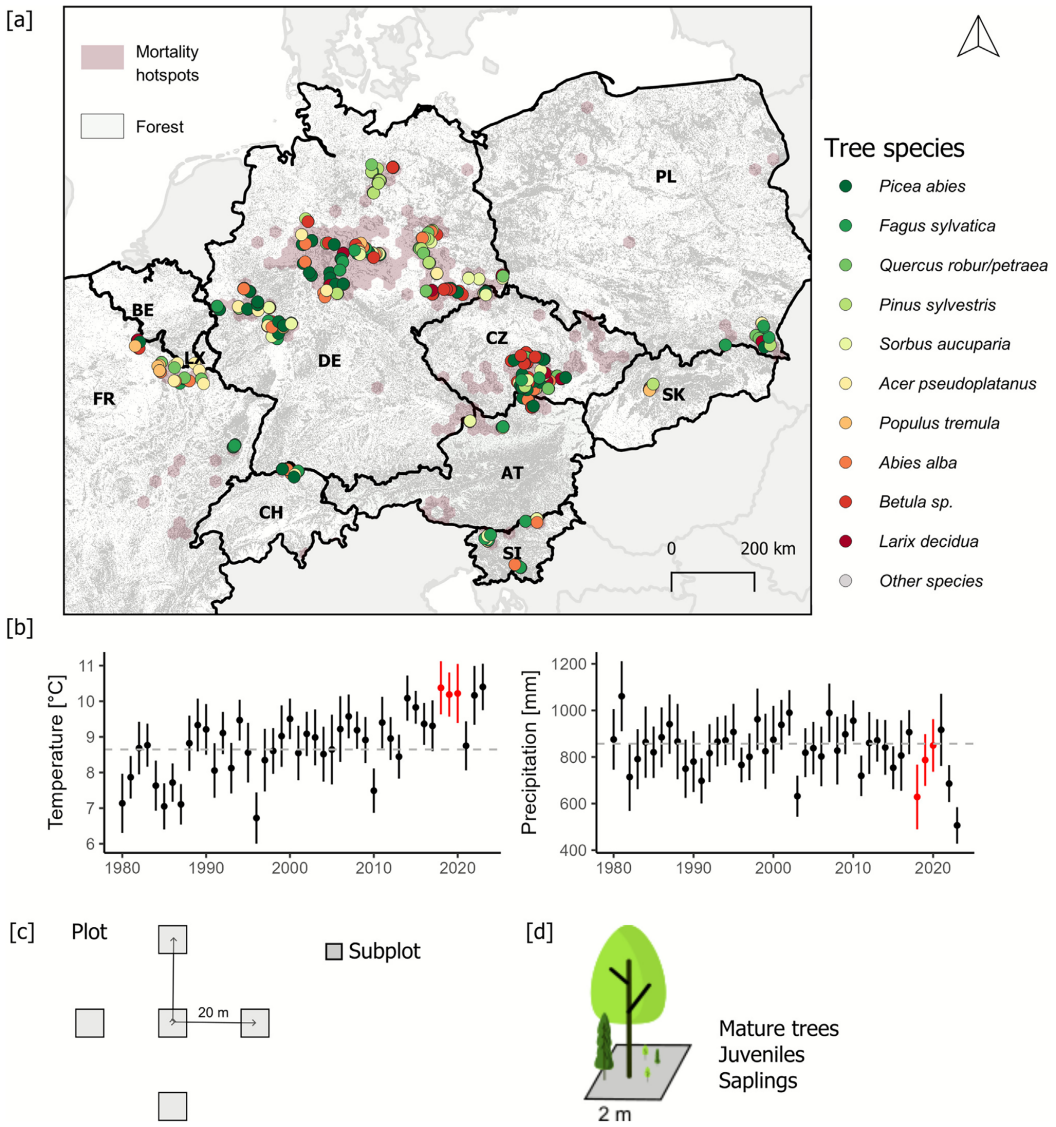
Study design, sampling scheme, and environmental context. (a) Geographic distribution of study plots (colored circles, *n* = 849) across ten European countries in tree mortality hotspots (pink hexagons) identified by remote sensing data. Plots are colored by their dominant tree species in the regeneration. (b) Time series of mean annual temperature (left) and annual precipitation sum (right) from 1980 to 2023. Data represents the median (dot) and interquartile range (whiskers) for the 849 sample locations. The disturbance years 2018–2020 are highlighted in red. (c) Sample plot design for surveying post‐disturbance forests, consisting of five subplots (4 m^2^ each) per plot, spaced 20 m apart in a regular orthogonal grid. (d) Tree sampling design within subplots, recording trees in three size classes that are, saplings (0.2–2 m in height), juveniles (> 2 m in height and < 10 cm at diameter at breast height, DBH), and mature trees (≥ 10 cm at DBH). AT = Austria, BE = Belgium, CH = Switzerland, CZ = Czech Republic, DE = Germany, FR = France, PL = Poland, SI = Slovenia, SK = Slovakia. Map lines delineate study areas and do not necessarily depict accepted national boundaries.

We used the European Forest Disturbance Map (Senf and Seidl 2021a) to identify hotspots of extensive tree mortality in the period 2018–2020. In the map, each pixel (resolution 30 m) holds information about the year of disturbance, disturbance agent and disturbance severity. To identify subcontinental‐scale disturbance hotspots, we aggregated disturbance information to hexagons (*d* = 25 km). We focused our analysis on temperate forests in Central Europe, here defined as the 10 countries Austria, Belgium, Czech Republic, France, Germany, Luxembourg, Poland, Slovakia, Slovenia, and Switzerland (Figure 1). We identified disturbance hotspots based on two complementary approaches. First, we aggregated all disturbance types to 25‐km hexagons to capture the full scope of disturbance activity. In a second step, we filtered for natural disturbance agents only—specifically, mortality caused by drought, fire, wind and bark beetle outbreaks—to exclude canopy openings from regular harvesting (Seidl and Senf 2024). Hotspots were defined as hexagons where the overall disturbance rate in 2018–2020 exceeded the 1986–2015 average by ≥ 300%, and where natural disturbances alone exceeded the historical average by ≥ 1000%. This approach accounts for the linked nature of planned and unplanned disturbances in European forests (Senf and Seidl 2021b). We note that natural disturbances in 2018–2020 were mainly dominated by drought and bark beetle outbreaks, with minimal windthrows in the selected countries. From these hotspots (see Figure 1) we opportunistically selected hexagons for field sampling, aiming to cover all major hotspot areas and countries affected across Central Europe. The average mean annual temperature (period 2018–2023) in the sampled disturbance hotspots was 9.9°C ± 0.9°C (mean ± SD, range: 7.2°C–12.0°C), and mean annual precipitation was 781 ± 187 mm (range: 527–1701 mm, Muñoz‐Sabater et al. 2021). Elevation ranged from 22 to 1261 m a.s.l., with a median of 415 m a.s.l.

Forest regeneration surveys were conducted within randomly selected disturbance patches, defined as contiguous areas of pixels classified as disturbed between 2018 and 2020 (Senf and Seidl 2021a). Disturbance patches were randomly selected from all patches meeting the minimum size criterion (> 0.5 ha). No additional stratification by slope or aspect was applied. Within each selected disturbance patch, we overlaid a systematic 20 m grid to identify candidate plot locations. The first subplot location was randomly chosen among grid intersections entirely contained within the selected disturbance patch. Each plot consisted of five 4‐m^2^ (2 × 2 m) subplots arranged in an orthogonal “+” pattern, with subplots spaced 20 m apart and oriented along the cardinal directions (Figure 1c). All five subplots were required to lie within the disturbed patch for a plot to be included; however, in small disturbance patches where the full “+” layout could not be accommodated, we accepted plots with four subplots (0.1% of all plots) and calculated all plot‐level metrics based on the actual sampled area. We selected a design of several small subplots over a single larger plot to better capture the spatial variation in tree regeneration on disturbed sites. The 4‐m^2^ subplot size approximately corresponds to the expected canopy area occupied by a single dominant tree in the stem exclusion stage—that is, at the end of the reorganization window, when crown competition begins to limit further stem recruitment (Franklin et al. 2007; Zeppenfeld et al. 2015). The minimum distance between two plots was 200 m. For each subplot, we collected tree information in three size cohorts: saplings (trees between 0.2 and 2 m in height), juveniles (trees > 2 m in height and ≤ 10 cm in diameter at breast height, DBH), and mature trees (> 10 cm in DBH). Saplings and juveniles are jointly referred to as tree regeneration throughout the text. We counted the number of stems per tree species and size category. For each plot, stem density (all species and species‐specific) was calculated as the total number of stems recorded across all subplots divided by the effective sampled area and scaled to 1 ha (stems ha^−1^). In total, we sampled 4245 subplots across 849 plots over 10 countries (Figure 1). In the analyses, we merged all plots collected in Luxembourg (*n* = 2) and Belgium (*n* = 3) with those collected in France (*n* = 58) due to small sample sizes per country.

### 2.2 Variables

To evaluate the structure and composition of post‐disturbed forests, we summarized tree information across vertical layers at the plot level. We focused the description of post‐disturbance forest conditions (i.e., our first research question) on four variables describing forest structure (i.e., stem density and vertical structure) and forest composition (tree species frequency and richness) (see Table 1 for details). All plot‐level structural metrics (i.e., stem density and vertical structure) were calculated across all plots, including those without tree regeneration. In tree species composition, we identified the ten most common species as the most frequently occurring ones (highest share of plots where a species occurs). Species‐specific stem densities were subsequently calculated as species abundance that is, conditionally on the species being present (excluding plots in which the species is absent).

**TABLE 1:** Indicators representing structure and composition, their units, and descriptions.

Category	Indicator	Unit	Description
Structure	Stem density	*n* ha^−1^	Sum of stems per ha, aggregated across tree species and vertical layers across all plots
	Vertical structure	Count	Number of vertical layers (i.e., saplings, juveniles and mature trees) present
Composition	Tree species frequency	Percent	Share of plots on which a tree species occurs
	Tree species richness	Count	Number of tree species per plot

Monthly temperature and precipitation data were obtained from the Copernicus ERA5‐Land reanalysis dataset at 9 × 9‐km spatial resolution (Grünig et al. 2024). We collected climatic data for 1980–2023 to characterize our study system (Figure 1c). We considered several climatic variables as potential predictors: mean annual variables and their anomalies relative to long term average conditions (reference period 1980–2015), and seasonality calculated variables as the coefficient of variation of monthly values for both temperature and precipitation. Additionally, we calculated standardized precipitation‐evapotranspiration indices (SPEIs) at two temporal scales: SPEI‐1 and SPEI‐12, representing the short term (SPEI‐1, 1 month scale) and long‐term (SPEI‐12, at 12 months' timescale) effects of drought. We collected soil physical characteristics relative to water holding capacity (clay and sand content) and available nitrogen as a proxy of access to nutrients (from Grünig et al. 2024). Soil data was available at a spatial resolution of 1 km^2^. We used the European Forest Disturbance Map (Senf and Seidl 2021a) to extract important disturbance characteristics for our study plots. Specifically, we calculated each plot's distance to the nearest undisturbed forest edge. Disturbance edges were derived from the 30‐m Landsat grid by identifying the boundary between pixels classified as disturbed (2018–2020) and those classified as undisturbed. Distance was measured as the Euclidean distance from the plot center to the edge of the nearest undisturbed pixel. We acknowledge that the 30‐m resolution and classification accuracy (mixed pixels) of the available disturbance data result in uncertainties regarding the exact location of the nearest undisturbed forests. Yet the data was well able to capture the general differences in the distance to potential seed sources for small and large disturbed patches. The median distance to the nearest forest edge was 60 m, with a range of between 30 and 242 m. Median disturbance severity—calculated as the percent of canopy removed in the disturbance—was 98.7%, ranging from 13% to 100% (Senf and Seidl 2021a). Overall, 75% of plots had very high disturbance severity (i.e., more than 99% of canopy removed). In total, we considered seven climatic variables, four soil variables, and two disturbance variables as potential predictors in the analysis.

### 2.3 Analyses

We used Generalized Additive Models (GAMs) to investigate the drivers of post‐disturbance regeneration density (*n* ha^−1^) using a Tweedie distribution to accommodate over‐dispersion and zero inflation (plots with no regeneration). To capture potential spatial structure, we included a spatial smoother based on plot coordinates. We also tested spatial autocorrelation between plots using Morans' *I*. We initially explored hierarchical mixed effect GAMs with country‐level random effects to account for unequal sample sizes and potential grouping effects. However, these random terms were not significant and did not improve model performance (Table S1 vs. Table S2) and were thus not retained in the subsequent analyses. Climate, soil and disturbance variables were included as smooth terms to capture non‐linear relationships. We initially assessed all candidate variables using univariate GAMs (18 models), ranked by Akaike's Information Criterion (AIC, Table S3) to guide screening. This allowed us to evaluate the relative importance of each predictor within its ecological group (i.e., climate, soil, and disturbance). Based on their univariate performance, ecological relevance, and low correlation (Figure S1), we selected the most biologically meaningful and robust predictors as the foundation for multivariate model building. Predictors were added sequentially, beginning with those showing the strongest univariate effects, followed by additional covariates chosen for their complementarity and independence.

We fitted both fixed‐effects‐only models and mixed‐effect models to evaluate whether hierarchical structure improved model performance. As the random effects did not add explanatory power, we report results from the fixed‐effects GAM. The final model (Table 2) included mean annual temperature and precipitation, disturbance severity, distance to the forest edge, soil clay content, and the interaction between temperature × precipitation—all of which had statistically significant and biologically interpretable effects on regeneration density. To assess the relative importance of individual predictors, we conducted drop‐one analyses by sequentially removing each term from the full GAM and comparing changes in AIC, log‐likelihood, adjusted *R* ^2^, and deviance explained. As the vast majority of our sites experienced high disturbance severity (70% with > 90% canopy removal), we conducted a sensitivity analysis to test whether the positive relationship between disturbance severity and regeneration density was also evident under lower‐severity disturbance conditions. To do so, we subset the data to include only plots with low to intermediate disturbance severity (< 70% canopy removal, corresponding to the 20th percentile of the severity distribution; *n* = 133) and refitted the GAM using the same model structure (see Figure S3).

**TABLE 2:** Selected final predictors of regeneration stem density. For the full list of predictors considered see Table S4.

	Predictors	Range and unit	Description
Climate	Precipitation	527–1701 mm	Annual precipitation sum
	Temperature	7.2–12.01°C	Mean annual temperature
Environment	Clay content	4%–51%	Clay content
	Available nitrogen	43–99 kg ha^−1^ year^−1^	Plant available nitrogen
Disturbance characteristics	Distance to edge	30–242 m	Distance between the plot center and the nearest edge of the undisturbed forest
	Severity	13%–100%	The % of the canopy removed in the disturbance

We subsequently focused our analysis on distinct patterns of post‐disturbance tree regeneration, particularly delayed and advanced regeneration. Delayed regeneration refers to plots with no trees in the regeneration layer (no saplings or juveniles present, Hansen et al. 2018), while sites with advanced regeneration had more than 1000 trees ha^−1^ taller than 2 m, indicating dense and well‐established regeneration (Seidl et al. 2024). This classification framework was used to explore how site conditions differ across regeneration patterns. Specifically, we compared site conditions between advanced and delayed regeneration using Wilcoxon rank sum tests.

We evaluated the short‐ and long‐term future prospect of post‐disturbance forests using two complementary modeling approaches: (i) forest simulation modeling and (ii) species distribution modeling. We used process‐based simulation modeling to better understand the short‐term development of disturbed sites. We simulated stem density development over the coming 30 years with the process‐based simulation model iLand, the individual‐based forest landscape and disturbance model (Rammer et al. 2024; Seidl et al. 2012). We specifically investigated convergence in stem density, as we expected that self‐thinning would reduce stem density in already densely stocked plots while infilling would increase stem densities in areas that are currently sparsely stocked. We used a two‐stage *k*‐means clustering approach to identify representative conditions for the simulation. Specifically, we first clustered plots based on their climatic and soil characteristics. Subsequently, within each environment cluster, we used our four response variables (Table 1) to identify groups with different stand structure and composition. This procedure resulted in a total of 12 distinct initializations for the simulation (each simulated as a 100 × 100 m stand), representing the full gradient of conditions observed across our plots. For each of these, we ran four climate change scenarios (present climate, RCP26, RCP45, RCP85) from three climate change models, repeating each run five times to account for stochasticity, and considering eight scenarios of seed input, resulting in a total of 5760 simulation runs. Given the importance of external seed input, we included eight different scenarios of seed input, ranging from increased seed availability to no seed scenarios and accounting for site‐specific factors such as proximity to undisturbed forests and local seed sources (see Figure S5). As output variable from the simulation model, we analyzed stem density over time.

Second, to understand the long‐term climate suitability of the currently present tree regeneration, we employed an existing species distribution modeling assessment (Wessely et al. 2024). The approach used is based on an ensemble of species distribution models (SDMs), predicting the continuous climatic suitability of tree species under future climate scenarios until the end of the 21st century. SDMs were trained using species occurrence data and climate variables to define each species' climatic niche at the level of 1‐km^2^ grid cells. A species was considered climatically suitable if the expected future climate remained within a species' climatic niche throughout the entire 21st century in decadal analysis time steps. Future projections were assessed under three Representative Concentration Pathways (RCP26, RCP45, and RCP85), representing mild, moderate, and severe climate change. We compiled climate suitability assessments for each species and plot location in our study. Specifically, we evaluated (i) the climatically suitable stem density, considering the portion of trees present where they remain climatically suitable in each climate change scenario, and (ii) the number of plots in which none of the currently present species are climatically suitable throughout the 21st century.

## 3 Results

### 3.1 Early Post‐Disturbance Tree Regeneration in Central European Temperate Forests

**FIGURE 2:**
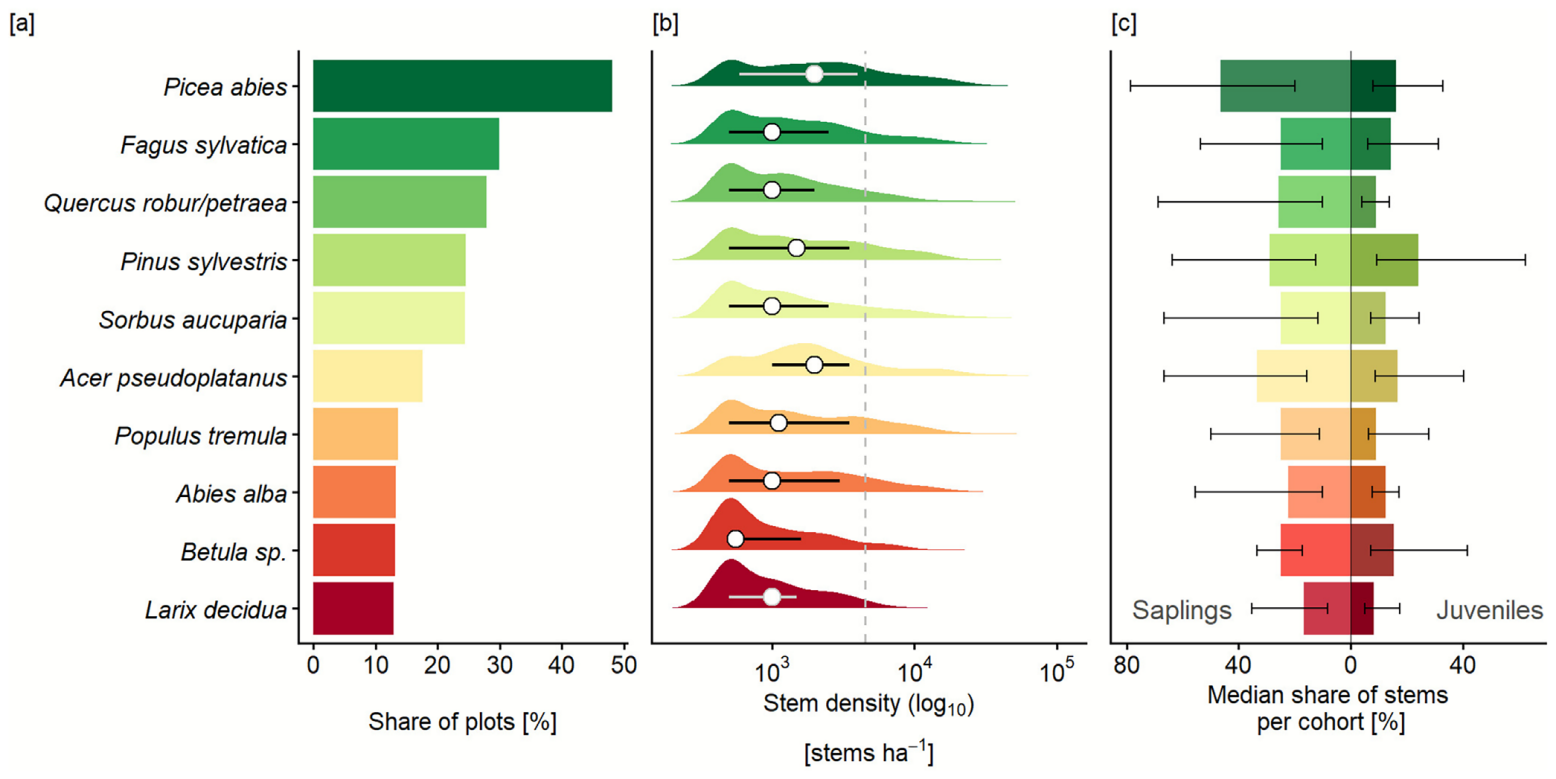
Early post‐disturbance tree species composition and structure in Central European forests. Shown are the top ten most frequent tree species based on species occurrence per plot. (a) Share of plots where each species occurs. (b) Distribution of species abundance (stem density, log_10_‐transformed). Curves show kernel‐density estimates; dots indicate species‐level medians and horizontal bars the interquartile range. Values only include plots where the species is present. Dashed line indicates the median stem density across all species. (c) Median share of total stem density contributed by each species in the sapling (0.2–2 m tall, left) and juvenile (> 2 m tall and < 10 cm in diameter at breast height, right) cohorts, with bars showing interquartile ranges.

Central Europe's temperate forests are highly resilient to the recent pulse of unprecedented disturbances. Overall stem density was high 3–5 years after disturbance, with a median number of trees on disturbed sites of 4750 *n* ha^−1^ (mean 7262 *n* ha^−1^, Table S5). The stem density distribution was highly positively skewed, indicating a tail of a few plots with very high densities (Figure 2, Table S5). The 10th percentile of the stem density distribution was 500 *n* ha^−1^, the 90th percentile 17,200 *n* ha^−1^, with a maximum stem density of 66,000 *n* ha^−1^. Forest structure post‐disturbance was relatively homogenous, with 47.3% of the plots being dominated by a single layer, whereof 94.9% were saplings (trees 0.2–2 m tall). Trees from all three vertical layers (saplings, juveniles, and mature trees) were present on only 7.9% of the plots. Sapling stem densities were generally higher than those in the juveniles' cohort (Figure 2c); of all the stems recorded, 84% were saplings, 14% juveniles, and 2% mature trees.

Overall, the most frequent tree species present after disturbance were *Picea abies* (on 48.1% of the plots), *Fagus sylvatica* (29.1%), *Quercus robur/petraea* (27.9%), *Pinus sylvestris* (24.5%), and *Sorbus aucuparia* (24.4%, see Figure 2a for the 10 most frequently recorded species, Table S6). *Picea abies* and *Acer pseudoplatanus* were the most abundant species (stem density of 1500 *n* ha^−1^ for both), followed by *Populus tremula* (1250 *n* ha^−1^). The maximum stem densities were observed for *Acer pseudoplatanus* (42,500 *n* ha^−1^), *Quercus robur/petraea* (42,000 *n* ha^−1^), and *Sorbus aucuparia* (36,000 *n* ha^−1^) (Figure 2b, Table S6). Overall, we recorded 35 tree species across the study area. Yet, tree species richness at the plot level was relatively low, with 19.1% of plots having only one tree species present, 44.3% having two or three tree species, and four or more tree species being present on 31.4% of plots.

### 3.2 Drivers of Post‐Disturbance Tree Regeneration

**FIGURE 3:**
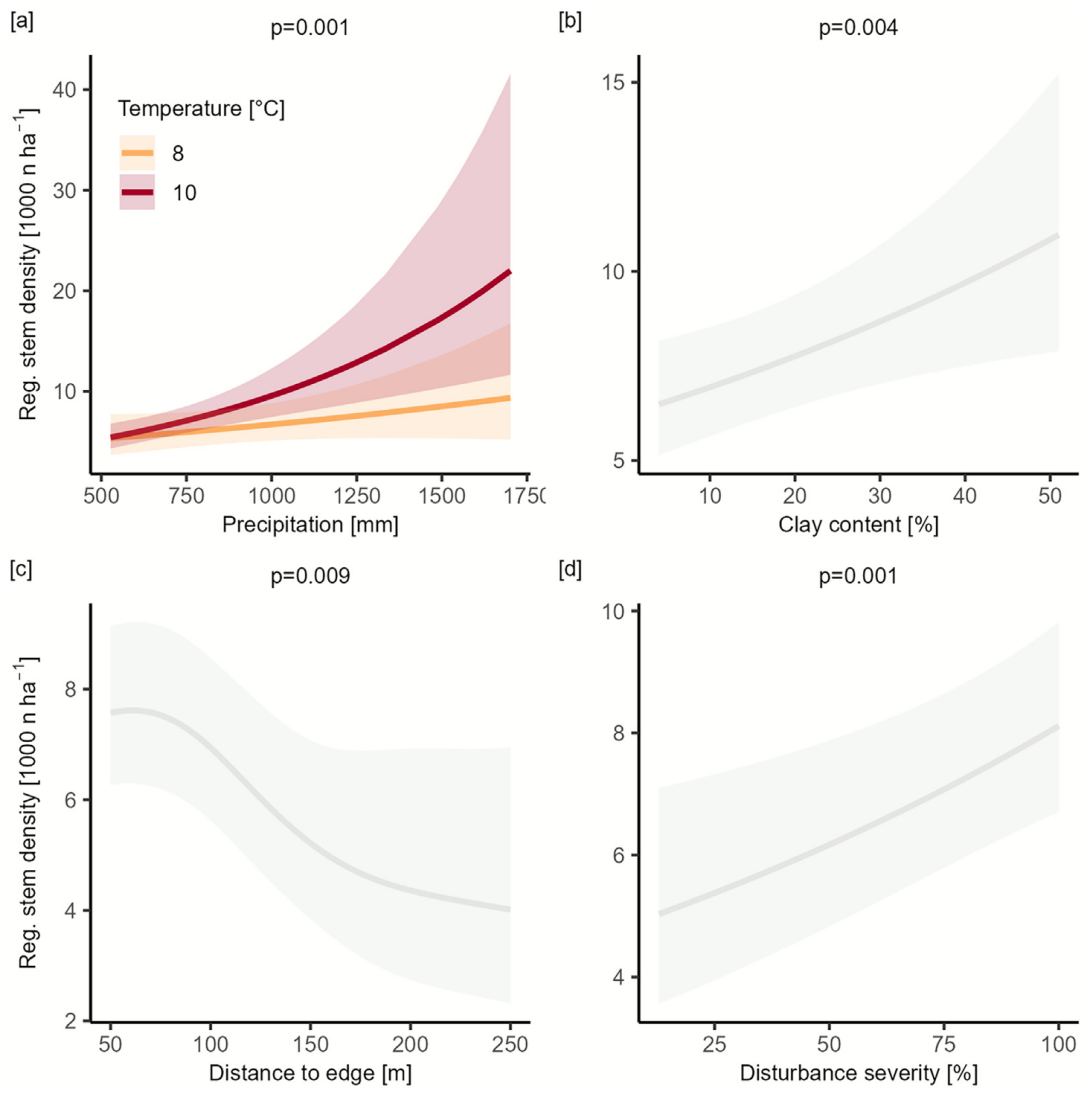
Effects of climatic, environmental and disturbance drivers on regeneration stem density. Generalized additive models (GAMs) were used to assess the partial effects of drivers of tree regeneration. (a) The interaction between mean annual precipitation sum and mean temperature in the years 2018–2023, (b) clay content, (c) distance to the edge of the undisturbed forest, and (d) disturbance severity. Solid lines represent estimated smooth effects, while shaded areas indicate 95% confidence intervals. *p*‐values at the top of each panel reflect the significance of the terms in the model.

Climatic, environmental, and disturbance variables all influenced regeneration stem density, with broadly similar contributions based on drop‐one model comparisons. Based on an analysis of the GAMs fitted to the data, each additional 100 mm of precipitation increased regeneration by approximately 9.3% on average (+811 *n* ha^−1^, *p* < 0.001). Both temperature (*p* = 0.006) and precipitation (*p* < 0.001) had significant and interactive effects on regeneration stem density. Notably, the positive effect on precipitation intensified at higher temperatures (interaction effect *p* = 0.001, Figure 3, Tables S1 and S2). Specifically, at a mean annual temperature of 10°C, the positive effect of precipitation on stem density was more than three times stronger than at 8°C. Regeneration stem density increased with clay content (as a coarse‐filter indicator for soil water holding capacity, *p* = 0.004). Furthermore, stem density increased with disturbance severity (*p* = 0.001) but decreased with distance from the forest edge (*p* = 0.009, Figures 3 and S2). At a distance to the undisturbed forest edge of 250 m, regeneration density dropped to 57% (4388 *n* ha^−1^, CI: 2518–7646) of the values expected 50 m from the edge (7762 *n* ha^−1^ CI: 6317‐9539). At a severity of 30%, predicted stem density was 5684 *n* ha^−1^ (95% CI: 4192–7708), while at 90% it was 7877 *n* ha^−1^ (95% CI: 6411–9679), representing an increase of 39%. Sensitivity analysis suggests that the positive severity effect observed in the full model is primarily driven by variation among high‐severity disturbance patches (Figure S3, Table S7). Nitrogen availability, soil depth, and sand content had no significant influence on stem density.

**FIGURE 4:**
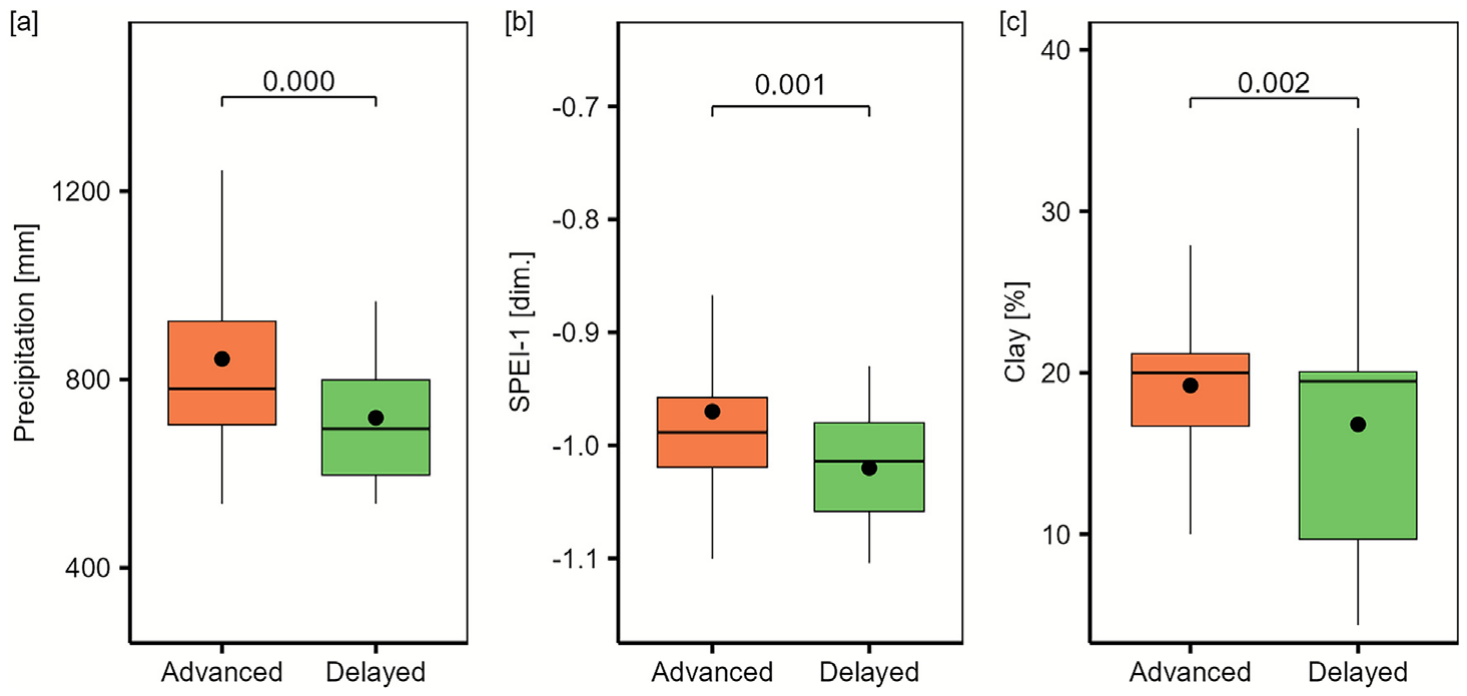
Differences in conditions between sites with delayed and advanced regeneration. (a) Mean annual precipitation sum 2018–2023, (b) Short‐term drought index (SPEI‐1) during the 2018–2020 drought period; (c) Soil clay content. Boxplots show the distribution of conditions across plots with delayed and advanced regeneration, with black dots indicating mean values, horizontal lines indicating median values, boxes giving the IQR, and whiskers extending to 1.5 × IQR. Shown *p*‐values were derived from Wilcoxon rank‐sum tests.

Overall, we found 6.7% (*n* = 57) of plots with delayed regeneration (no regeneration present) and 26.7% (227) of plots with advanced regeneration (> 1000 stems ha^−1^ of > 2 m height), with the remaining plots exhibiting intermediate regeneration patterns. Sites with delayed regeneration had lower mean annual precipitation levels (710 vs. 784 mm, *p* = 0.002, Wilcoxon rank sum test), were more strongly exposed to drought in the years 2018–2020 (Standardized Precipitation‐Evapotranspiration Index (SPEI‐1): −1.01 vs. −0.99, *p* = 0.001), and had a lower ability to store water and nutrients in the soil (i.e., lower clay content) than those featuring advanced regeneration (Figure 4). Disturbance characteristics, temperature, and climate anomalies did not differ significantly between sites with advanced and delayed regeneration (Figure S4).

### 3.3 Future Outlook for Recently Disturbed Sites

Process‐based model simulations suggest that all disturbed sites will be able to regain forest cover. Specifically, simulations suggest gradual infilling for sites that currently exhibit delayed regeneration, while for sites that already hold high stem densities self‐thinning will set in. These two processes eventually led to a reduction in the overall variation in stem density and a convergence at relatively high stem densities (between 6197 and 7136 *n* ha^−1^) 30 years after disturbance (Figure S5). These results were consistent across climate change scenarios.

**FIGURE 5:**
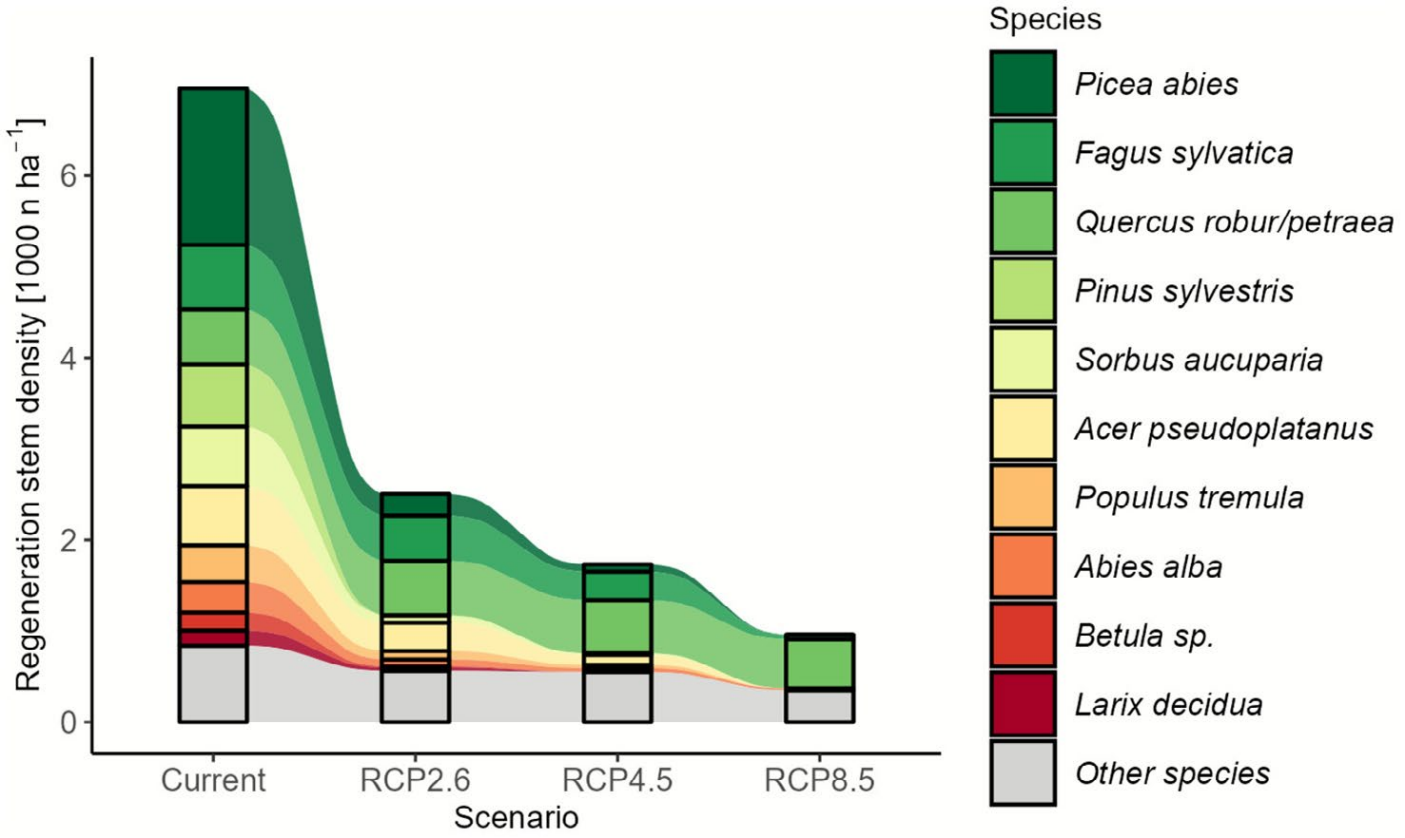
Future climate suitability of current tree regeneration on post‐disturbance sites across Central Europe. Average stem density of currently present species (explicitly shown for the 10 most common species, with all other species combined to “other”) that remain climatically suitable throughout the 21st century under mild (RCP2.6), moderate (RCP4.5), and severe (RCP8.5) climate change. The “current” column gives the stem density and species distribution as recorded in the field in 2023. Note that values under future climate scenarios are not projections of future regeneration but rather an assessment of the future climate suitability of the currently present regeneration.

Species distribution models (Wessely et al. 2024) suggest that species currently present on disturbed sites in Central Europe are largely not climatically suitable under the conditions expected for the 21st century (Table 3, Figure 5). On average, only between 36.0% (under mild climate change, RCP2.6) and 13.8% (under severe climate change, RCP8.5) of the currently present tree regeneration will remain within their climatic niche over the course of the 21st century. On 38.5% to 61.7% of plots (for RCP2.6 and RCP8.5, respectively), none of the currently regenerating trees are climatically suitable throughout the 21st century (see Tables 3 and S8 for country‐level analysis). The most common maladapted species are *Picea abies* (not climatically suitable on 369 plots under RCP4.5, which is 93% of its current occurrence), *Sorbus aucuparia* (*n* = 203, 98% of current occurrence), and *Fagus sylvatica* (*n* = 246, 63%). In contrast, species generally well‐adapted to the climate expected for the 21st century at the sites they regenerated are *Quercus robur/petraea* (*n* = 232, i.e., 94% of current occurrence), *Carpinus betulus* (*n* = 90, 91%), and *Populus* sp. (*n* = 23, 69%). However, all species except *Quercus robur/petraea* are increasingly maladapted with increasing severity of climate change (RCP8.5), particularly *Fagus sylvatica* (a decrease of 58% of climatically suitable plots between RCP2.6 and RCP8.5), *Fraxinus excelsior* (decrease by 39%), and *Abies alba* (decrease by 28%). This trend of decreasing climatic suitability with increasing severity of climate change was consistent across all sampled locations (Tables 3 and S8).

**TABLE 3:** Future climate suitability of current tree regeneration on post‐disturbance sites in temperate forests across 10 Central European countries. Shown are (a) the average shares of tree species stems per plot that remain within their climatic niche until the end of the 21st century under three climate change scenarios (RCP26, RCP45, RCP85), and (b) the average share of plots where none of currently present species are projected to remain within their climatic niche until the end of the century. Values are shown as the average shares (%) of stems (left columns) and the number of plots (right columns). Belgium and Luxemburg were pooled with France due to small individual sample sizes. Twenty‐first century climatic suitability of a species for a given site was assessed following Wessely et al. (2024), with a species being considered climatically suitable only if it is projected to remain within its climatic niche throughout the entire 21st century. Note that these values do not represent tree regeneration across the entire country, but only pertain to the sampled disturbance hotspots (Figure 1).

Country	(a) Average plot‐level share of currently present species that remain within their climate niche until 2100 (% of current stems per plot)			(b) Share of plots where none of the currently present species in the regeneration remain within their climate niche until 2100 (% of all plots)		
	RCP26	RCP45	RCP85	RCP26	RCP45	RCP85
Austria	54.7	38.2	11.8	15.8	29.8	66.7
Czech Republic	28.8	10.6	4.7	34.8	65.2	71.1
France	46.5	39.8	19.2	19.0	19.0	49.2
Germany	26.0	18.7	13.4	41.0	49.2	55.1
Poland	36.0	22.1	23.7	18.9	40.5	29.7
Slovakia	45.7	15.4	9.8	5.6	50.0	50.0
Slovenia	61.9	42.9	14.2	8.9	12.5	44.6
Switzerland	48.3	42.9	20.5	16.7	12.5	33.3
Total	36.0	24.9	13.8	32.2	44.4	55.5

## 4 Discussion

We found high disturbance resilience of Central European forests following the unprecedented high‐severity disturbance pulse in 2018–2020. The presence of tree regeneration on 93% of plots 3 to 5 years after disturbance supports our hypothesis on the robust regeneration capacity of Central European forests (H1). Robust post‐disturbance recovery resulted from the combination of strong post‐disturbance regeneration and a considerable presence of advanced regeneration, that is, the legacy of the pre‐disturbance forest (Bače et al. 2015; Petrovska et al. 2023; Seidl et al. 2024). We note that the importance of advanced regeneration is contingent on disturbance type: the main disturbances investigated here—drought and bark beetle mortality—predominately affect trees in the upper canopy, with trees in the understory often surviving the disturbance and contributing to forest recovery. In fire‐driven systems, for example, such advanced regeneration would be consumed by fire (Dey and Hartman 2005) and would thus not support forest recovery in a similar way.

Our findings underscore that post‐disturbance tree regeneration is strongly contingent on climatic and disturbance conditions (Thom et al. 2023; Young et al. 2019). Specifically, lower levels of precipitation significantly reduce post‐disturbance recovery, while warmer temperatures increase stem density when precipitation is sufficient (H2, Figure 3). Disturbance severity and size (here quantified via the distance to the nearest undisturbed forest edge) influenced tree regeneration in distinctly different ways: Stem density increased with disturbance severity (Figure 3d) and decreased with increasing distance from the forest edge (Figure 3c). These opposing effects suggest that while strong canopy openings can create favorable conditions for regeneration by increasing light availability, a greater distance to seed sources (i.e., larger disturbance patches) likely limits regeneration due to reduced seed availability (Shive et al. 2018; Wild et al. 2014). Furthermore, both factors may contribute to more extreme microclimatic conditions (Máliš et al. 2023; Thom et al. 2023) but the net outcome for regeneration depends on whether beneficial effects (e.g., light availability) outweigh negative ones (e.g., moisture stress or seed limitation). The species composition of the regeneration was dominated by drought‐sensitive conifers such as *Picea abies* and by pioneer species ( *Populus tremula* , *Betula* spp.), while relatively more drought‐tolerant broadleaves such as *Quercus* spp. and *Fagus sylvatica* were less abundant (Figure 2, see Niinemets and Valladares 2006 for relative drought tolerance). The dominance of species with relatively high water demands suggests that increasingly frequent hotter‐drought conditions (IPCC 2023) may challenge the currently establishing forests in the future. Furthermore, our analysis of drivers of regeneration underlines that dry conditions are a strong limiting factor of tree regeneration (Beloiu Schwenke et al. 2023; Hansen et al. 2018; Pozner et al. 2022).

Important limitations must be acknowledged when interpreting our results. First, by using remote sensing to identify disturbance hotspots for sampling (Senf and Seidl 2021a), we focused primarily on stand‐replacing disturbances. However, low‐ and mixed‐severity disturbances are also common in Central Europe (Čada et al. 2013; Meigs et al. 2017) (e.g., following recent drought in *Fagus sylvatica* forests [Seidl et al. 2024]), yet were not in the focus of our study. Second, we surveyed sites only 3 to 5 years after disturbance, meaning that the window of reorganization may not yet have been closed on most sites. Plots with no regeneration thus may indicate delayed regeneration rather than regeneration failure. To overcome this limitation, we complemented our analyses of empirical data with process‐based model simulations, indicating that even sites with no or low regeneration will eventually return to being forested (Figures S4 and S5), supporting our hypothesis H3. Third, we found that clay content (i.e., a proxy for high soil water holding capacity) was the only soil variable that had a significant effect on regeneration density (Figure 3). We note, however, that more soil variables such as nutrient availability and pH value can have important effects on tree regeneration (Kramer et al. 2014; Liu 2025), yet the coarse resolution of the subcontinental datasets used here (Grünig et al. 2024, resolution of 1 km^2^) may have prevented such local effects from being captured. Additionally, an important factor influencing post‐disturbance tree regeneration is the stand structure and composition prior to disturbance (Wild et al. 2014). Unfortunately, no consistent pre‐disturbance stand information was available across the 10 countries investigated here, which is why this important driver could not be considered in our analyses. Furthermore, the vast majority of forests in Central Europe are managed for a variety of ecosystem services. Hence, our findings are the result of both natural regeneration processes (i.e., seed dispersal, germination, establishment) and management actions (e.g., salvage logging, planting, browsing protection). These interventions vary across countries and may affect regeneration outcomes, potentially contributing to differences in species composition (Seidl et al. 2024) and regeneration success. Consequently, the influence of the effects of post‐disturbance management warrants further investigation. Finally, we note that our analysis on future climate suitability only addresses the currently present tree regeneration. Future establishment of trees that are potentially better adapted to the emerging climate conditions, for example, through natural or assisted migration (Gustafson et al. 2023; Leech et al. 2011) or epigenetic mechanisms (i.e., with the next generation being better adapted than the previous one [Miryeganeh and Armitage 2025]) were not considered here. Therefore, our findings represent a conservative estimate, not considering potentially increased adaptive capacity of the forest at the genetic and species level.

We found that the majority of the currently regenerating trees are not climatically suitable under projected 21st century climate (Figure 5). This finding provides two important insights: First, it highlights that the current composition of forests strongly lags behind climatic conditions, and even recently established trees are not able to track the exceedingly high pace of climate change (Rosenblad et al. 2023; Thom et al. 2022). This could result from limited seed availability of climate‐adapted species, or from managers planting species that they worked with in the past (despite not necessarily being well‐adapted to emerging future conditions). As tree mortality in 2018–2020 particularly affected *Picea abies* forests (Schuldt et al. 2020), the fact that the most common species found in the regeneration was again *Picea abies* suggests high inertia, or potentially even a lock‐in of the tree species composition. Our findings are in line with research from the western United States, showing that natural forest adaptation is approximately 10 times slower than the current rate of climate change (Rosenblad et al. 2023; Serra‐Diaz et al. 2018). Future analyses with simulation models could help explore how long this inertia may persist and how shifts in species composition will unfold under continued disturbance and climate change. Second, our results suggest that significant further shifts in species composition are needed to maintain climate suitability, as many currently present trees are increasingly maladapted to the climate emerging in the 21st century. Recent work from naturally developing forests indicates that structural diversity can partly mitigate the effects of a lock‐in of the species composition (Sommerfeld et al. 2021). Yet, structural diversity on our sampled plots was relatively low, suggesting no strong signal of increased structural diversity shortly after disturbance (Gough et al. 2022; Seidl et al. 2024).

Our findings have important implications for management. First, the high degree of maladaptation underscores that proactive adaptation strategies are essential to ensure climate‐adapted forests, as both natural regeneration processes and the slow uptake of new management approaches in forestry hamper the ability of the system to keep pace with rapidly changing conditions. Second, it highlights that tending measures favoring climate‐adapted species wherever they are present on post‐disturbance sites are an important means of supporting climate‐adapted forests. While *Picea abies* was the most common species found in the regeneration (present on 48% of plots), pure *Picea abies* regeneration was only found on 5.7% of plots. This underlines that there is considerable potential for future silvicultural measures like selective thinning to reduce the degree of maladaptation to future climate. Despite concerns about the future outlook of the currently established trees, our findings of robust regeneration suggest that ecosystem functions and services can be maintained on disturbed sites in the near future, underlining the current resilience of Central Europe's forests to disturbance (Cerioni et al. 2024; Seidl et al. 2024; Senf and Seidl 2022). The currently established tree regeneration can serve as a transitional cohort towards forests that are better adapted to the emerging climate conditions. We conclude that disturbances offer critical opportunities for reorganization towards more climate‐adapted forests. Forest policy and management should recognize these opportunities in future disturbance events and plan to utilize them towards accelerated climate adaptation.

## Author Contributions

**Mária Potterf:** data curation, formal analysis, investigation, methodology, visualization, writing – original draft, writing – review and editing. **Christian Schattenberg:** data curation, investigation, project administration. **Kirsten Krüger:** data curation, investigation, writing – review and editing. **Kilian Hochholzer:** data curation, investigation, writing – review and editing. **Werner Rammer:** conceptualization, funding acquisition, investigation. **Marc Grünig:** investigation, writing – review and editing. **Kristin H. Braziunas:** investigation, writing – review and editing. **Christina Dollinger:** investigation, writing – review and editing. **Aikio Erhardt:** investigation. **Jean‐Claude Gégout:** investigation, writing – review and editing. **Lisa Geres:** investigation, visualization. **Sina Greiner:** investigation. **Tomáš Hlásny:** investigation, writing – review and editing. **Anne Huber:** investigation. **Jonas Kerber:** investigation. **Judit Lecina‐Diaz:** investigation, writing – review and editing. **Lisa Mandl:** investigation. **Roman Modlinger:** investigation. **Johannes S. Mohr:** investigation, writing – review and editing. **Jörg Müller:** investigation. **Miguel Muñoz Mazón:** investigation, writing – review and editing. **Paulina E. Pinto:** investigation, writing – review and editing. **Tobias Richter:** investigation. **Sebastian Seibold:** investigation. **Cornelius Senf:** investigation. **Josep M. Serra‐Diaz:** investigation, writing – review and editing. **Ana Stritih:** investigation, writing – review and editing. **Dominik Thom:** investigation, writing – review and editing. **Alba Viana‐Soto:** investigation. **Jiayun Zou:** investigation, writing – review and editing. **Rupert Seidl:** conceptualization, funding acquisition, investigation, methodology, writing – review and editing.

## Conflicts of Interest

The authors declare no conflicts of interest.

## Supporting information


**Data S1:** gcb70734‐sup‐0001‐Supinfo.pdf.

## Data Availability

The data that support the findings of this study are openly available in EU_forest_regeneration at https://doi.org/10.5281/zenodo.18449661.
