# Supplementary material for: Tree Regeneration After Unprecedented Forest Disturbances in Central Europe Is Robust but Maladapted to Future Climate Change
Source: Glob Chang Biol. 2026 Feb 6;32(2):e70734. doi: 10.1111/gcb.70734 (PMC12881712; doi:10.1111/gcb.70734)
Supplement: Supplementary file 1 — Data S1: gcb70734‐sup‐0001‐Supinfo.pdf. [file GCB-32-e70734-s001.pdf]

# Supplementary Information

## Supplementary figures

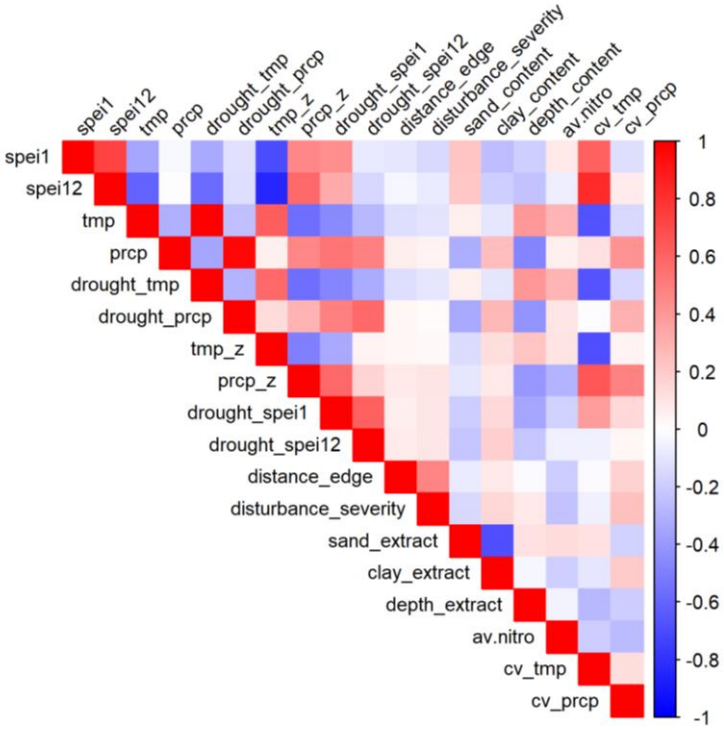


*Figure S 1* ***Spearman correlation matrix of predictor variables used in the analysis.*** *The plot shows pairwise Spearman rank correlations among predictor variables. Only the upper triangle is displayed, with color gradients indicating the strength and direction of monotonic relationships (red = positive, blue = negative). Acronyms:* ***tmp*** *= temperature,* ***prcp*** *= precipitation,* ***cv*** *= coefficient of variation (seasonality),* ***_z*** *= standardized anomalies (z-scores),* ***SPEI1/12*** *= Standardized Precipitation Evapotranspiration Index over 1/12 months. Variables prefixed with* ***drought_*** *refer to the 2018–2020 drought period; all others represent the 2018–2023 baseline.*

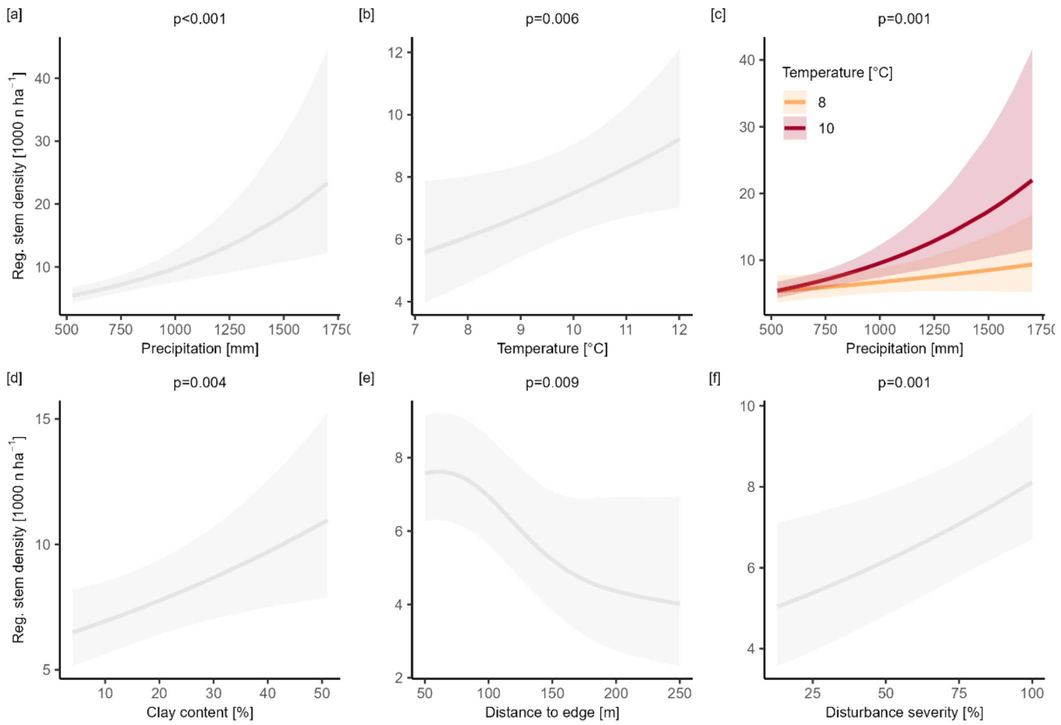


*Figure S 2: Effects of climate, environmental and disturbance characteristics on regeneration stem density. Generalized additive models (GAMs) were used to assess the partial effects of key effects on regeneration stem density. Effect of (a) precipitation, (b) temperature, (c) their interaction, (d) clay content, (e) the distance to the forest edge and (f) disturbance severity. Solid lines represent estimated smooth effects, while shaded areas indicate 95% confidence intervals. P-values at the top of each panel reflect the significance of the interaction terms.*

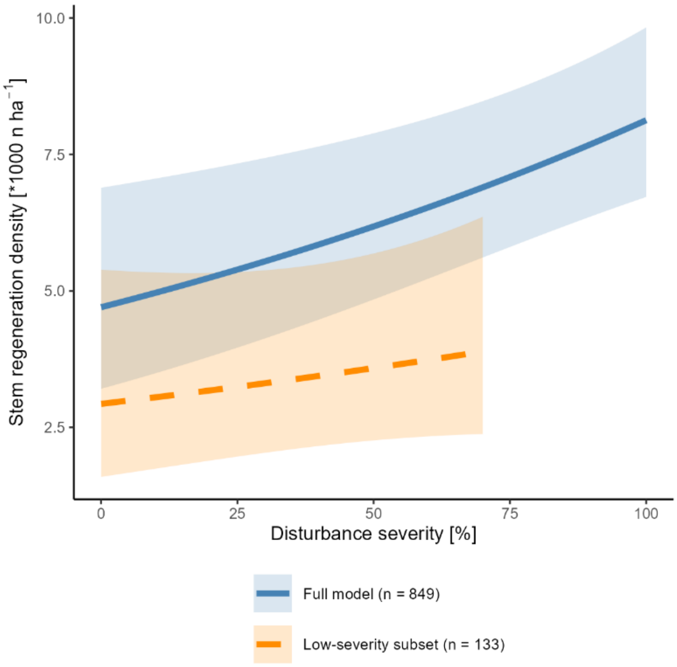


*Figure S 3: Predicted effect of intermediate and high- disturbance severity on post-disturbance tree stem regeneration density (n $ha^{-1}$), based on Generalized Additive Models (GAMs). The solid line shows predictions from the full dataset (n = 849), while the dashed line shows results from a subset including only intermediate and low-severity plots (disturbance severity <70%, n = 133). Shaded areas represent confidence intervals. Regeneration density increases with disturbance severity in the full model, but this effect weakens and becomes statistically uncertain in the low-severity subset, indicating that the positive relationship is largely driven by high-severity disturbance dynamics.*

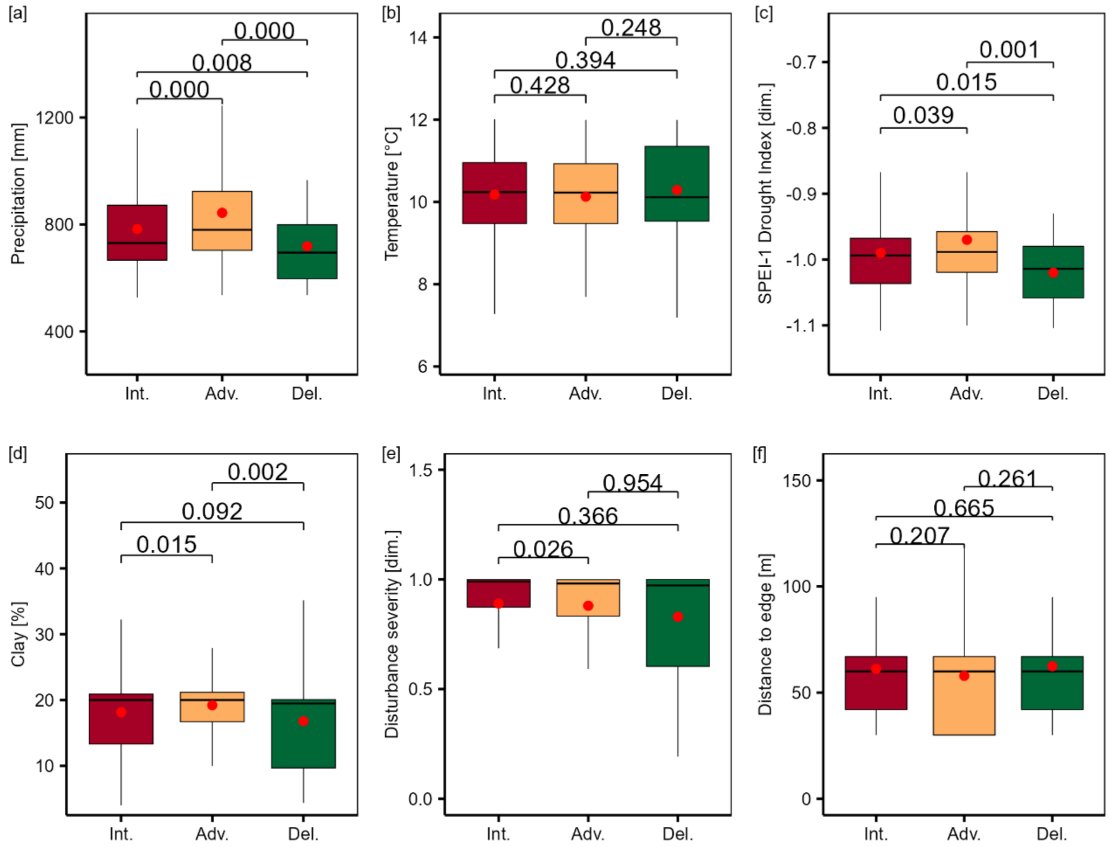


*Figure S 4 Climatic, environmental and disturbance characteristics drivers of regeneration stem density under different regeneration statuses. (a) Mean annual precipitation sum over the study period, (b) mean annual temperature, c) short term drought index (SPEI-1) during the 2018–2020; (d) clay content [%], (e) disturbance severity and (f) distance to edge [m]. Boxplots show the distribution of values across delayed, other, and advanced regeneration statuses, with red dots indicating mean values. Show are p-values, representing pairwise comparisons derived from the Wilcoxon rank-sum test. Del – Delayed, Int. – Intermediate, Adv. – Advanced regeneration.*

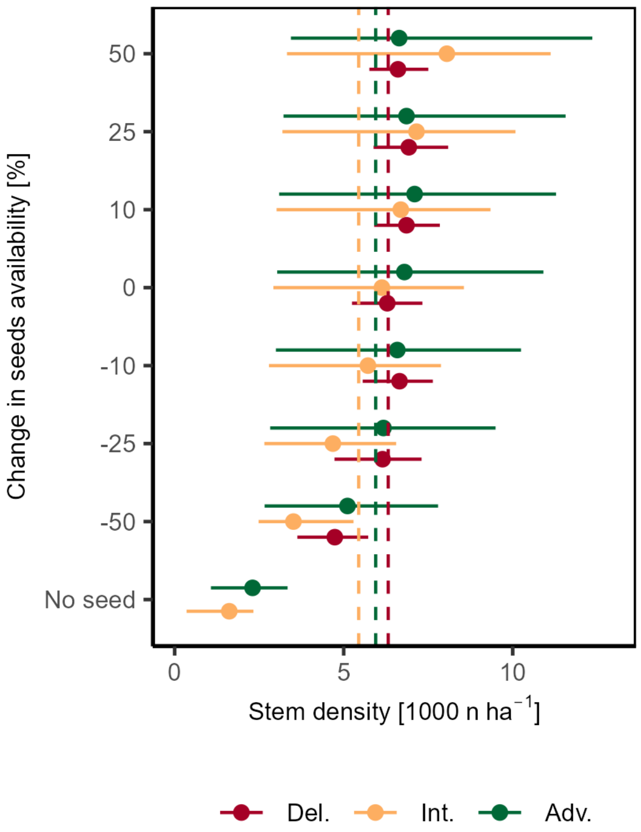


*Figure S 5* ***Sensitivity of regeneration stem density to changes in seed availability across regeneration types.*** *Simulated stem densities (points: median; lines: interquartile range) in relation to different seed availability scenarios ranging from no seed to +/-50% increase or reduction in seed availability, shown separately for Delayed (Del.), Intermediate (Int.), and Advanced (Adv.)regeneration types. Each data point summarizes median stem density across simulation years 25–30 for a given seed availability scenario (-50%, -25%, 0%, 25%, 50%,), expressed relative to the baseline (0% change). The baseline reflects expected seed input based on proximity to existing forest and surrounding tree cover. Dashed vertical lines indicate the mean stem density for each regeneration type. Note that no seed scenario in Delayed is empty. This represents an unlikely scenario, where no seeds are expected to come within the simulation period (30 years).*

# Supplementary tables

*Table S 1 Summary table of the fixed effects GAM model for regeneration stem density*

| | Explained Deviance: 10.89% | | |
|---|---|---|---|
| *Predictors* | *Estimates* | *CI* | *p* |
| (Intercept) | 6975.94 | 6540.66 – 7440.19 | **<0.001** |
| s(prcp) | | | **<0.001** |
| s(tmp) | | | **0.006** |
| s(distance_edge) | | | **0.009** |
| s(disturbance_severity) | | | **0.001** |
| s(clay_extract) | | | **0.004** |
| s(av.nitro) | | | 0.333 |
| ti(disturbance_severity,distance_edge) | | | 0.794 |
| ti(prcp,tmp) | | | **0.001** |
| s(x,y) | | | **<0.001** |
| Observations | 849 | | |
| $R^2$ | 0.093 | | |

*Table S 2 Summary table of the fixed and random effects GAMM model for regeneration stem density*

| | Explained Deviance: 10.78% | | |
|---|---|---|---|
| *Predictors* | *Estimates* | *CI* | *p* |
| (Intercept) | 6793.72 | 6384.94 – 7228.68 | **<0.001** |
| s(prcp) | | | **<0.001** |
| s(tmp) | | | **0.001** |
| s(distance_edge) | | | **0.011** |
| s(disturbance_severity) | | | **0.001** |
| s(clay_extract) | | | **0.002** |
| s(av.nitro) | | | 0.360 |
| ti(disturbance_severity,distance_edge) | | | 0.734 |
| ti(prcp,tmp) | | | 0.156 |
| s(country_pooled) (Random Effect) | | | 0.241 |
| s(x,y) | | | **<0.001** |
| Observations | 849 | | |
| $R^2$ | 0.094 | | |

*Table S 3 Results from univariate Generalized Additive Models (GAMs) used as an initial screening to rank the importance of candidate predictors based on Akaike Information Criterion (AIC). Variables with lower AIC values indicate stronger univariate explanatory power for regeneration stem density. While several predictors showed relatively strong effects (e.g., drought-related indices and soil variables), only mean annual temperature (tmp), annual precipitation (prcp), and their interaction were retained in the final multivariate model based on ecological relevance, predictor independence, and overall model performance.*

| Predictor | AIC |
|---|---|
| drought_prcp | 16008.0 |
| prcp | 16027.7 |
| spei3 | 16031.7 |
| av.nitro | 16035.0 |
| drought_spei12 | 16037.8 |
| drought_spei1 | 16039.1 |
| clay_extract | 16041.3 |
| tmp | 16046.5 |
| depth_extract | 16051.1 |
| distance_edge | 16052.3 |
| disturbance_severity | 16052.6 |
| cv_tmp | 16053.5 |
| spei1 | 16054.3 |
| cv_precipitation | 16054.8 |
| drought_tmp | 16056.5 |
| spei12 | 16056.9 |

*Table S 4 Full list of predictors of the regeneration stem density that were used as the input data for univariate model to identify the most important predictors of stem regeneration density.*

| | Predictors | Description |
|---|---|---|
| Climate | Precipitation | Sum precipitation per year |
| | Temperature | Mean annual temperature |
| | Precipitation (growth season) | Sum precipitation per growth season/year. |
| | Temperature (growth season) | Mean temperature growth season/year. |
| | Precipitation (seasonality) | Coefficient of variation of the monthly precipitation per year. |
| | Temperature (seasonality) | Coefficient of variation of the monthly temperatures per year. |
| | SPEI1 | Standardized Precipitation-Evapotranspiration Index (1 month) |
| | SPEI12 | Standardized Precipitation-Evapotranspiration Index (12 months) |
| Environment | Clay content | Clay content |
| | Sand content | Sand content |
| | Available nitrogen | Plant available nitrogen |
| Disturbance characteristics | Distance to edge | Euclidean distance between the plot and the nearest forest edge |
| | Severity | The % of the removed mature trees |

*Table S 5 The distribution of the post-disturbance indicators for structure and composition*

| *Indicators* | *Unit* | *0* | *0.01* | *0.25* | *0.5 (median)* | *0.75* | *0.9* | *1* | *mean* |
|---|---|---|---|---|---|---|---|---|---|
| Stem density | $n\ ha^{-1}$ | 0 | 0 | 2000 | 4750 | 9500 | 17500 | 66000 | 7262 |
| Vertical layers | # | 0 | 0 | 1.0 | 1.0 | 2.0 | 2 | 3 | 1.5 |
| Occurrence | | 0 | 0 | 0.5 | 0.7 | 0.9 | 1 | 1 | 0.7 |
| Richness | | 0 | 0 | 2.0 | 3.0 | 4.0 | 5 | 13 | 2.8 |

*Table S 6 Summary of species-specific stem density across 849 forest regeneration plots. For each species, the table reports: (1) total number of recorded stems, (2) plot occurrence as count and percentage of plots where the species was present, (3) conditional stem density statistics (minimum, maximum, mean ± standard deviation, and median with interquartile range) based on plots where the species was present, and (4) unconditional stem density, calculated as average stem density per species across all plots (including absences). This represents the species' landscape-level contribution to regeneration. Species are listed by Latin name; "sp." indicates identification to genus level only. Table is ordered by decreasing share of plots per species[%].*

| | | | *Conditional stem density [n/ha]* | | | *Unconditional stem density [n/ha]* |
|---|---|---|---|---|---|---|
| *Species* | *Total stems [#]* | *Plot count [#] (share [%])* | *Min-Max* | *Mean* ± SD | *Median (IQR)* | *Mean* |
| *Picea abies* | 2948 | 408 (48.1) | 500 - 31000 | 3612 ± 4651 | 2000 (3406) | 1736 |
| *Fagus sylvatica* | 1250 | 254 (29.9) | 500 - 20000 | 2461 ± 3278 | 1000 (2000) | 736 |
| *Quercus robur/rubra* | 1042 | 237 (27.9) | 500 - 42000 | 2199 ± 3920 | 1000 (1500) | 614 |
| *Pinus sylvestris* | 1198 | 208 (24.5) | 500 - 34000 | 2880 ± 3902 | 1500 (3000) | 706 |
| *Sorbus aucuparia* | 1110 | 207 (24.4) | 500 - 36000 | 2681 ± 4406 | 1000 (2000) | 654 |
| *Acer pseudoplatanus* | 1114 | 149 (17.6) | 500 - 46000 | 3737 ± 6048 | 2000 (2500) | 656 |
| *Populus tremula* | 692 | 116 (13.7) | 500 - 39000 | 2982 ± 4714 | 1125 (3000) | 407 |
| *Abies alba* | 578 | 113 (13.3) | 500 - 22500 | 2555 ± 3412 | 1000 (2500) | 340 |
| *Betula sp.* | 348 | 112 (13.2) | 500 - 17500 | 1551 ± 2186 | 562 (1125) | 205 |
| *Larix decidua* | 288 | 110 (13) | 500 - 9500 | 1309 ± 1337 | 1000 (1000) | 170 |
| *Carpinus betulus* | 480 | 91 (10.7) | 500 - 17000 | 2637 ± 3361 | 1000 (2750) | 283 |
| *Pseudotsuga menziesii* | 128 | 59 (6.9) | 500 - 4500 | 1081 ± 858 | 625 (1000) | 75 |
| *Pinus strobus* | 92 | 33 (3.9) | 500 - 8500 | 1386 ± 1815 | 500 (1000) | 54 |

| | | | | | | |
|---|---|---|---|---|---|---|
| *Prunus avium* | 69 | 30 (3.5) | 500 - 6000 | 1150 ± 1212 | 500 (1000) | 41 |
| *Alnus glutinosa* | 64 | 25 (2.9) | 500 - 3500 | 1280 ± 925 | 1000 (1000) | 38 |
| *Salix caprea* | 40 | 24 (2.8) | 500 - 2500 | 833 ± 545 | 500 (500) | 24 |
| *Populus sp.* | 63 | 23 (2.7) | 500 - 5000 | 1370 ± 1058 | 1000 (750) | 37 |
| *Fraxinus excelsior* | 343 | 19 (2.2) | 500 - 17000 | 9026 ± 5141 | 8500 (0) | 202 |
| *Sorbus aria* | 42 | 17 (2) | 500 - 5000 | 1235 ± 1264 | 500 (500) | 25 |
| *Ulmus sp.* | 78 | 17 (2) | 500 - 7500 | 2294 ± 2215 | 1500 (2500) | 46 |
| *Acer platanoides* | 115 | 14 (1.6) | 500 - 23000 | 4107 ± 5900 | 2000 (2875) | 68 |
| *Quercus sp.* | 42 | 14 (1.6) | 500 - 7000 | 1482 ± 1722 | 1000 (938) | 24 |
| *Tilia sp.* | 52 | 14 (1.6) | 500 - 9500 | 1857 ± 2468 | 1000 (375) | 31 |
| *Acer campestre* | 21 | 10 (1.2) | 500 - 3000 | 1050 ± 864 | 500 (875) | 12 |
| *Castanea sativa* | 37 | 8 (0.9) | 500 - 6000 | 2297 ± 2169 | 1250 (3594) | 22 |
| *Fraxinus ornus* | 11 | 7 (0.8) | 500 - 1500 | 786 ± 393 | 500 (500) | 6 |
| *Alnus incana* | 19 | 6 (0.7) | 500 - 4500 | 1583 ± 1563 | 1000 (1375) | 11 |
| *Robinia pseudoacacia* | 62 | 6 (0.7) | 500 - 28750 | 5208 ± 11533 | 500 (0) | 37 |
| *Ostrya carpinifolia* | 9 | 4 (0.5) | 500 - 3000 | 1125 ± 1250 | 500 (625) | 5 |
| *Ilex aquifolium* | 6 | 3 (0.4) | 1000 - 1000 | 1000 ± 0 | 1000 (0) | 4 |
| *Juglans regia* | 2 | 2 (0.2) | 500 - 500 | 500 ± 0 | 500 (0) | 1 |

| | | | | | | |
|---|---|---|---|---|---|---|
| *Sorbus torminalis* | 2 | 2 (0.2) | 500 - 500 | 500 ± 0 | 500 (0) | 1 |
| *Juniperus sp.* | 1 | 1 (0.1) | 500 - 500 | 500 ± NA | 500 (0) | 1 |
| *Salix sp.* | 1 | 1 (0.1) | 500 - 500 | 500 ± NA | 500 (0) | 1 |
| *Tilia cordata* | 1 | 1 (0.1) | 500 - 500 | 500 ± NA | 500 (0) | 1 |

*Table S 7: Summary of the GAM for tree regeneration density (n = 133) fitted to a subset of low and intermediate severity plots (disturbance severity <70%). The model explained 38.47% of deviance (adjusted $R^2 = 0.34$). While disturbance severity was not a significant predictor in this subset ($p = 0.155$), regeneration density was significantly related to soil clay content ($p = 0.026$), the interaction between temperature and precipitation ($p = 0.001$), and spatial structure ($p < 0.001$). This highlights the importance of microclimatic factors in driving regeneration under low and intermediate-disturbance severity.*

| | **Explained Deviance: 38.47%** | | |
|---|---|---|---|
| *Predictors* | *Estimates* | *CI* | *p* |
| (Intercept) | 5713.46 | 4545.24 – 7181.95 | **<0.001** |
| s(prcp) | | | 0.216 |
| s(tmp) | | | 0.885 |
| s(distance_edge) | | | 0.166 |
| s(disturbance_severity) | | | 0.155 |
| s(clay_extract) | | | **0.026** |
| s(av.nitro) | | | 0.329 |
| ti(disturbance_severity,distance_edge) | | | 0.598 |
| ti(prcp,tmp) | | | **0.001** |
| s(x,y) | | | **<0.001** |
| Observations | 133 | | |
| $R^2$ | 0.340 | | |

*Table S 8* ***Future climate suitability of current tree regeneration on post-disturbance sites across Central European countries.*** *The table presents two key indicators of projected climatic suitability of regenerating tree species under future climate scenarios (RCP2.6, RCP4.5, and RCP8.5):(a) the average number of tree species per plot that are projected to remain within their climatic niche throughout the 21st century (richness, with associated percentage of stems in parentheses), and (b) the number and share of plots where none of the currently present tree species remain suitable (lost, shown as count (percentage)).Values represent (a) the average species richness per plot and country, with the percentage in parentheses indicating the average proportion of current species retained under each climate scenario, (b) the number of post-disturbance plots per country where no currently present species remain climatically suitable, with the share of such plots shown in parentheses. The "Total" row summarizes values across all countries. Richness values are calculated as weighted averages based on the number of plots per country. The lost values reflect absolute counts and percentages across the full dataset. Belgium and Luxembourg are pooled with France due to limited sample sizes in those countries. Climatic suitability was assessed following Wessely et al.[12]; a species was considered suitable if it is projected to remain within its 21st-century climatic niche for the entire period.*

| | *(a) Average plot-level share of currently present species that remain within their climate niche until 2100 (% of current richness per plot)* | | | | *(b) Share of plots where none of the currently present species in the regeneration remain within their climate niche until 2100 (% of all plots)* | | | |
|---|---|---|---|---|---|---|---|---|
| *Country* | *Richness (average)* | *RCP26* | *RCP45* | *RCP85* | *Plot (count)* | *RCP26* | *RCP45* | *RCP85* |
| Austria | 3.4 | 1.6 (54.7) | 1.1 (38.2) | 0.4 (11.8) | 57 | 9 (15.8) | 17 (29.8) | 38 (66.7) |
| Czech Republic | 3.4 | 1 (28.8) | 0.4 (10.6) | 0.3 (4.7) | 135 | 47 (34.8) | 88 (65.2) | 96 (71.1) |
| France | 3.6 | 1.7 (46.5) | 1.6 (39.8) | 0.9 (19.2) | 63 | 12 (19) | 12 (19) | 31 (49.2) |
| Germany | 3.0 | 0.9 (26) | 0.7 (18.7) | 0.5 (13.4) | 459 | 188 (41) | 226 (49.2) | 253 (55.1) |
| Poland | 3.5 | 1.4 (36) | 0.9 (22.1) | 1.1 (23.7) | 37 | 7 (18.9) | 15 (40.5) | 11 (29.7) |
| Slovakia | 3.6 | 2 (45.7) | 0.5 (15.4) | 0.5 (9.8) | 18 | 1 (5.6) | 9 (50) | 9 (50) |
| Slovenia | 4.5 | 2.6 (61.9) | 2 (42.9) | 1 (14.2) | 56 | 5 (8.9) | 7 (12.5) | 25 (44.6) |
| Switzerland | 5.1 | 2.5 (48.3) | 2 (42.9) | 1.5 (20.5) | 24 | 4 (16.7) | 3 (12.5) | 8 (33.3) |
| Total | 29.0 | 20 (36) | 19 (24.9) | 14 (13.8) | 849 | 273 (32.2) | 377 (44.4) | 471 (55.5) |

98
